# Economic evaluations and their use in infection prevention and control: a narrative review

**DOI:** 10.1186/s13756-018-0327-z

**Published:** 2018-02-27

**Authors:** Elissa Rennert-May, John Conly, Jenine Leal, Stephanie Smith, Braden Manns

**Affiliations:** 1 Departments of Medicine and Community Health Sciences, University of Calgary, and Alberta Health Services, AGW5 Ground Floor SSB, 1403 29 St NW, Calgary, AB T2N 2T9 Canada; 2 Departments of Medicine, Immunology, Microbiology and Infectious Diseases, Pathology and Laboratory Medicine, O’Brien Institute for Public Health and Snyder Institute for Chronic Diseases, University of Calgary and Alberta Health Services, Calgary, AB Canada; 3 Department of Community Health Sciences, University of Calgary and Infection Prevention and Control, Alberta Health Services, Foothills Medical Centre, Calgary, AB Canada; 4 Department of Medicine, University of Alberta and University of Alberta Hospital and Alberta Health Services, Edmonton, AB Canada; 5 Departments of Medicine and Community Health Sciences, O’Brien Institute for Public Health and Libin Cardiovascular Institute, University of Calgary and Alberta Health Services, Calgary, AB Canada

## Abstract

**Background:**

The objective of this review is to provide a comprehensive overview of the different types of economic evaluations that can be utilized by Infection Prevention and Control practitioners with a particular focus on the use of the quality adjusted life year, and its associated challenges. We also highlight existing economic evaluations published within Infection Prevention and Control, research gaps and future directions.

**Design:**

Narrative Review.

**Conclusions:**

To date the majority of economic evaluations within Infection Prevention and Control are considered partial economic evaluations. Acknowledging the challenges, which include variable utilities within infection prevention and control, a lack of randomized controlled trials, and difficulty in modelling infectious diseases in general, future economic evaluation studies should strive to be consistent with published guidelines for economic evaluations. This includes the use of quality adjusted life years. Further research is required to estimate utility scores of relevance within Infection Prevention and Control.

## Background

Health-care associated infections (HAI) are common. In Canada approximately 200,000 patients will develop a HAI each year with 8000 associated deaths [1]. In the United States (US), there are over two million HAI annually [2]. HAIs are also extremely costly, with the overall annual direct costs to hospitals in the US ranging from $35.7 to $45 billion [3, 4]. There are numerous Infection Prevention and Control (IPC) interventions that can be utilized in hospitals to prevent HAI and their spread [5]. IPC activities include programs such as surveillance, hospital investigations when there are outbreaks, measures to prevent spread of contagious organisms, education for healthcare employees, patients and family members, and reporting of HAI to national organizations [6].

While many IPC programs and activities can be justified given that HAIs result in patient morbidity and lengthy hospital admissions [7], not every activity or program within IPC should be funded. When determining which programs should be implemented, the efficiency and effectiveness of such programs must be considered. Economic evaluations can determine which IPC strategies are cost-effective and provide reasonable value for money [7]. We sought to conduct a narrative review of the various types of economic evaluations, recommendations by national institutions, and factors to consider for economic evaluations, particularly the use of quality adjusted life years (QALYs) within IPC and their associated challenges.

## Economic evaluations

All economic evaluations assess value for money by comparing the impact of competing interventions on both costs and consequences simultaneously [8]. There are a variety of types of economic evaluations described briefly below.

### Cost-minimization analysis

A basic form of analysis is cost-minimization analysis which is used when the clinical effectiveness of two interventions are the same - so the choice between them relates to their relative costs [8]. For example, if two programs to promote hand hygiene were identical in terms of efficiency than only the comparisons of costs would be relevant.

### Cost-effectiveness analysis

A cost-effectiveness analysis (CEA) compares consequences using natural clinical units such as life years gained, or infections avoided. The advantage to CEAs is that they are easier to conduct and the consequences are simple to understand clinically, however it can be difficult to compare the results of different evaluations if the same measure of clinical outcome is not used (for instance, how to compare a study reporting a cost per pneumonia avoided with a study reporting a cost per heart attack prevented) [8]. An example of a CEA is a recent study which compared universal and targeted decolonization for methicillin resistant *Staphylococcus aureus* (MRSA) in patients in the intensive care unit, to screening and then isolating patients who were colonized with MRSA [9]. It was more cost-effective (e.g. lower cost and prevented more MRSA infections) to complete universal and targeted decolonization compared to screening and isolation [9].

### Cost-utility analysis

A cost-utility analysis (CUA) is an extension of a CEA, where the measure of health benefit is a measure that considers both length and quality of life. This is often represented by the QALY, calculated by multiplying the utility (a measure of preference for a person’s overall quality of life) of a given health state by the time spent in that health state [8]. The QALY allows for direct comparison between economic evaluations comparing different types of interventions and health conditions, rendering cost per QALY estimates more comparable. For example, a study compared the cost per QALY of rectal culture-guided antibiotic prophylaxis with standard ciprofloxacin prophylaxis [10]. The culture guided group saved 0.0002 QALYs and $24 per patient, by preventing more infections [10].

### Cost benefit analysis

Cost-benefit analysis (CBA) is another form of economic evaluation where all consequences, including clinical outcomes, are expressed in monetary terms. CBAs can be a challenge in health care evaluations because many of the health benefits can be difficult to quantify in monetary terms, though they can be helpful when there are non-health benefits of interventions that require inclusion (for instance, the benefits of receiving health care locally vs travelling or when there are other process benefits that wouldn’t be quantified through the usual QALY rubric) [8]. In a recent cost-benefit analysis of a supplementary measles immunization program in a highly immunized population [11], the authors noted that the management of 187 cases of measles cost $864,000 (hospitalization costs, case management and earnings lost). In order for supplemental vaccination to be considered cost-effective, they estimated that the vaccination would need to cost less than $66 to $1877 per patient (depending on different scenarios) [11]. As the authors concluded that such a program would be unlikely to exceed these costs, supplementary measles immunization was considered cost-effective [11].

## Guidelines for conducting economic evaluations

The most recent guidelines for the conduct of economic evaluations from the Canadian Agency for Drug and Technologies in Health were published in 2017 [12]. These guidelines are meant to improve the quality of economic evaluations for health technologies, and to ensure comparability across different analyses. They recommend that a CUA be utilized with the outcomes expressed as QALYs. Any other type of economic evaluation must be justified.

The International Society for Pharmacoeconomics and Outcomes Research (ISPOR) also has guidelines on the reporting of economic evaluations which suggest the use of QALYs whenever possible, and are used widely in Canada, the United States and internationally [13].

## Estimating and using the quality adjusted life year

As described above, a CUA is frequently the recommended type of economic evaluation and the outcome that is suggested for use by all contemporary guidelines is the QALY. A QALY is calculated by multiplying the utility (a measure of preference for a person’s overall quality of life – usually varying on a scale from 0 to 1, where 1 represents perfect health and 0 is equivalent to death) for a given health state by the time spent in that health state [14]. Utility scores can be determined in a variety of ways. They can be measured directly – through use of the visual analogue scale, the time trade-off and the standard gamble [14], or they can be measured indirectly, through questionnaires like the Euroqol EQ-5D, or the Health Utilities Index.

In the visual analogue scale participants with a certain health condition are presented with a scale ranging from worst to best imaginable health state and on that scale they place where they feel their current health state is [15]. This provides subjective weights and an ordinal ranking of health outcomes, but it does not invoke the notion of trade-off [15]. In the time trade-off method individuals have a choice between living the rest of their life (*t*) in a particular health state (*i*) or living for a shorter time period (*x*) but in perfect health. The time is varied until the participant feels ambivalent about the two options and then the preference score for *i* is *x*/*t* [15]. In the standard gamble individuals have to choose between the certainty of remaining in a given health state *i* and an alternative with two outcomes of perfect health with the probability *p* and death with the probability 1-*p.* The *p* is varied until the participant is indifferent between the two choices and then the score for state *i* for time *t* is *p* [15]. If a participant places a higher value on state *i* then a higher probability of perfect health will be needed for the individual to be indifferent between *i* and the gamble of having perfect health [15]. In both cases a score between 0 and 1 results, with higher scores reflecting better overall quality of life.

As the above direct methods can be time consuming and challenging for patients, several instruments have been developed that can generate utilities from scores on a variety of domains, for instance the EQ-5D, the SF-6D and the health utilities index [14]. These generic measures are used for valuing health related quality of life based on health status within certain areas [14]. The responses to these questionnaires are then converted into a single utility value.

We illustrate the use of QALYs with an example from the IPC literature which compared different IPC programs designed to prevent surgical site infections [16]. The authors used hypothetical data to estimate how surgical site infections would impact morbidity and mortality and the influence of these infections could be measured in QALYs. Two different scenarios were represented, in the first scenario patients either did or did not develop an infection and those who did develop an infection died shortly after surgery. The patients who did not develop an infection had 7.575 more QALYs than those who did acquire an infection. In another scenario, a patient develops an infection but recovers and after several months improves to the same health state as a patient who never develops an infection. This patient has 7.475 QALYs after surgery [16].

## Economic evaluations within infection prevention and control

### Positive and negative economic evaluations

Health care systems universally work within the confines of cost containment and it can be difficult to reconcile programs such as IPC interventions that improve patient quality of care with their associated costs. Economic evaluations can enhance the evidence base demonstrating how certain IPC programs, despite their expense, improve care for patients in a cost-effective manner and in some cases can result in cost savings. For example, a Canadian study demonstrated that after an IPC program became regionalized with standardized policies and procedures, with a budget of $6.7 million over the four years, the program resulted in cost savings of $9.1 million with a reduction of 4739 HAI cases [17]. It is evidence such as this that justifies the existence and continued funding for health care quality improvement programs such as IPC. Alternatively, economic evaluations will also demonstrate when IPC programs are not cost-effective. A study from the United States looked at the effectiveness of universal screening for MRSA to prevent hospital acquired MRSA infections [18]. The authors determined that while the rate of detection of MRSA was higher in universal screening, it was also more costly and did not significantly reduce the rate of hospital acquired MRSA infections compared to targeted screening. Therefore, no implementation of a universal screening program was advised [18].

### Is a full economic evaluation required?

A systematic review from 2005 examining 70 different studies which performed economic analyses of HAI [2], a systematic audit of economic evidence linking HAI and IPC interventions from 1990 to 2000 [19], and a recent systematic review from 2016 [20], all found that frequently only partial economic evaluations were completed. Simple cost analyses of infection were commonly utilized, guidelines for economic evaluations were not followed, and the quality of reporting according to ISPOR was low [2, 19, 20]. This likely reflects that health economics in IPC is still a relatively new area.

If a strategy that improves outcomes also saves money in the short-term, then there is potentially no need for a complete economic evaluation assuming proper methodology was utilized. However, if there is additional cost associated with the intervention then other factors do need to be considered, such as infections prevented, or the impact on QALYs, allowing for comparison between different interventions [8].

## Use of quality adjusted life years in economic evaluations within infection prevention and control

With respect to the important question of whether to invest in IPC programs, a question to be asked is: what are the benefits in terms of improvements to patient’s quality of life and survival, as well as the impact on overall costs? The purpose of IPC interventions is to prevent infections thus improving patient outcomes and resulting in additional QALYs [7]. Without randomized controlled trials (RCTs), this can be difficult to assess as the economic evaluation needs to be able to estimate what care for patients would have cost if they had not developed any infection [21].

The study used previously to describe the calculation of QALYs examined a model with six different IPC programs designed to prevent surgical site infections using infection related costs and QALYs [16]. The authors considered the outcomes of the cost of the IPC program, the cost of infection to the hospital, the cost to the community services and the patient-borne costs of the infection. The different IPC programs had varied upfront costs but some had more cost-savings, due to more infections prevented – resulting in different incremental QALY estimates. This reinforces that just assessing the change in costs related to preventing infection does not fully answer the question posed by their study. Changes in health benefits must be considered [16]. The authors then determined which IPC strategy had the lowest cost per QALY, and their conclusions contrast with the decisions that might be made when only considering the evaluation from the viewpoint of costs spent and saved [16].

In another recent study, the authors created a HAI cost-effectiveness policy model simulating elderly patients admitted to the intensive care unit (ICU). The objective was to determine the cost-effectiveness of hospitals’ continuing investments in HAI prevention in ICUs. Subsequently, multiple health states following the ICU admission were considered including bloodstream infection related to an access line, ventilator associated pneumonia, the conditional probability of inpatient deaths due to each specific HAI type, as well as incremental hospital costs associated with each infection [22]. A five year time horizon was used, which included QALYs and healthcare costs (dependent on whether or not a HAI developed) [22]. Published literature was used to determine the costs and rates of HAI and Medicare data was used to estimate the monthly conditional probabilities of health states for those who had or had not developed a HAI [22]. The authors determined that by continuing to use an IPC program to prevent HAI in ICUs, it would cost $14,250 per life year gained and $23,277 per QALY gained (US dollars) compared to not using an IPC program [22]. While not cost-saving, the IPC program to prevent HAI was still considered cost-effective [22].

## Challenges with quality adjusted life years and economic evaluations in infection prevention and control

A systematic review of CUA related to Infectious Diseases including IPC was published in 2005 [23], noting only 122 CUA publications over a 21 year time period. There are many reasons for this, including a focus by payers on costs, a lack of RCTs in IPC, the complexity of modelling in infectious diseases, and few estimates for utility measures in IPC. In general, hospitals implementing IPC programs care about the costs spent and saved, and considering costs on their own is a simple accounting exercise.

There are few RCTs in IPC, and utilities as well as costs and benefits are frequently determined alongside these trials. This has likely impacted the number of informative CUAs in IPC. The lack of RCTs may in part be due to the difficulty in comparing the multitude of different IPC interventions, rendering it extremely difficult to complete one succinct RCT [16]. Therefore, in order to model the costs and benefits of IPC programs, multiple sources of information may need to be funneled into one model, creating a more difficult modelling scenario [16].

In addition to the issues just described, modelling for infectious diseases in general can be quite complex and requires specific expertise [23]. For example, the lack of an IPC program may lead to a patient developing an infection with antimicrobial resistant bacteria via transmission from another patient causing adverse consequences and increased costs. However, it can be difficult to model this continued transmission.

The estimation of utilities in infectious diseases appears to be challenging for several reasons. Indeed, studies that have estimated utilities in infectious diseases have noted a very wide interquartile range compared to 13 other disease categories [23]. The broad range of utilities available may at first glance render a CEA, which examines costs per life year gained or per infection avoided, more appealing to conduct.

Another limitation of using a QALY in HAI, which are typically transient [24], is that it can be difficult to determine a trade-off between quality and quantity of life [25]. Transient health states are those that last for a specific brief time, often less than one year, followed by a return to full health [26]. While traditional methods for measuring quality of life may not be appropriate for these transient health states, there are techniques adapted from the conventional methods which can be utilized [26]. While it could be argued that with such a short health state any influence on QALYs would be minimal, there may be longer lasting effects from even a short-term impact on health such as infection. In studies looking at health valuation specifically in transient health states such as dentistry and infection following hip arthroplasty, valuation of the health state can still be accomplished which subsequently can be turned into QALYs allowing for comparison between programs [27, 28].

## Conclusions

The majority of economic evaluations in IPC are partial evaluations only considering costs. Given finite resources and fixed budgets, conducting rigorous economic evaluations of IPC programs will help policy makers understand where and how to spend scarce healthcare dollars. Utilizing a CUA and QALYs will allow comparison to other programs where healthcare dollars might be spent and ensure that resources are being used in sustainable and cost-effective programs.

The utilization of the QALY is not without its difficulties. This necessitates the use of more complex models and many of the necessary pieces of information, including utilities and time spent in particular health states lack reliable information within the realm of IPC.

Despite these challenges, given that IPC interventions were designed to improve patient safety and quality of care it is appropriate to not only consider costs saved from the hospital standpoint during admission but also the benefit to health through the use of QALYs.

Future economic evaluations in the area of IPC interventions should aim to follow rigorous guidelines for economic evaluations and justify when they are not used. CEAs that demonstrate cost savings and improved outcomes can inform funding decisions, but in most situations, use of the QALY when comparing different IPC interventions should be carefully considered.

Finally, the difficulty with estimating utilities in infectious diseases, in the context of this review, and in particular IPC should be addressed through additional studies that collect utilities for different health states related to HAI [23].

As more health economists work alongside infectious diseases and IPC specialists, it is likely that the outcomes relevant to health economics will be included, making economic evaluations more common. Collaborating with health economists can help in addressing the difficulties in uncertainty and amalgamating data when there are multiple sources of information and a lack of RCTs. Additionally, they can aid in creating valid economic models that take into account the transient nature of HAI and encourage the use of proper techniques to obtain utilities and subsequent QALYs.

## Abbreviations

CEA: Cost Effectiveness Analysis
CUA: Cost Utility Analysis
HAI: Healthcare Associated Infections
ICU: Intensive Care Unit
IPC: Infection Prevention and Control
ISPOR: International Society for Pharmacoeconomics and Outcomes Research
MRSA: Methicillin Resistant *Staphylococcus aureus*
QALY: Quality Adjusted Life Year
US: United States
